# Supplementary material for: Interplay between competitive and cooperative interactions in a three-player pathogen system
Source: R Soc Open Sci. 2020 Jan 22;7(1):190305. doi: 10.1098/rsos.190305 (PMC7029927; doi:10.1098/rsos.190305)
Supplement: Supplementary material for: Interplay between competitive and cooperative interactions in a three-player pathogen system [file rsos190305supp1.pdf]

Supplementary material for: Interplay between competitive and cooperative interactions in a three-player pathogen system

Francesco Pinotti[1], Fakhteh Ghanbarnejad[2,3,4], Philipp Hövel[2,5], and Chiara Poletto[1,*]

[1]INSERM, Sorbonne Université, Institut Pierre Louis d'Épidémiologie et de Santé Publique, IPLESP, F75012, Paris, France
[2]Institut für Theoretische Physik, Technische Universität Berlin, Hardenbergstraße 36, 10623 Berlin, Germany
[3]The Abdus Salam International Centre for Theoretical Physics (ICTP), Trieste, Italy
[4]Physics Department, Sharif University of Technology, P.O. Box 11165-9161, Tehran, Iran
[5]School of Mathematical Sciences, University College Cork, Western Road, Cork T12 XF62, Ireland
[*]chiara.poletto@inserm.fr

1 Methods

Mean-field dynamics

We studied the mean-field dynamical system described by equations (2.1) in the main paper by stability analysis and numerical integration. We derived closed-form expressions for all fixed-points and almost all conditions underlying their local stability. We used numerical evaluation of the Jacobian's spectrum to study stability whenever an analytical solution was not possible and to check the accurracy of the analytical results as well. We then numerically integrated the mean-field equations exploring different initial conditions. Numerical integration of the ordinary differential equations was performed in *Python 3.6* using the function *odeint* from the *Scipy* package.

Mean-field dynamics with communities

The epidemic in each population follows the same dynamics as in equations (2.1) of the main text. The infection terms, however, must be modified in order to account for the different contributions (between and within community) to the force of infection. Specifically, the force of infection due to, e.g. B_1, acting on an individual in community $c = 1, 2$ becomes $\beta_1 \left[(1-\epsilon)X_1^{(c)} + \epsilon X_1^{(c')}\right]$, where $c \neq c'$. The two distinct terms appearing in this expression represent the contributions due to infected individuals in the same community (c) and in the other community (c'), respectively. Notice that for 2 interacting populations, as considered in the main paper, we can reduce the number of independent equations from 12 to 10 by exploiting density conservation within each population, i.e. $\sum_Z Z^{(c)} = 1$, where $Z^{(c)}$ denote the fraction of individuals in state Z and community $c = 1, 2$. Numerical integration of these equations is performed in the same way as in the case of a single population.

Network models

We used the algorithm outlined in [1] to efficiently generate Erdős-Rényi networks. In order to generate modular networks with n_C communities and adjustable community strength, we first group nodes into n_C different communities. Here we chose for simplicity to assign exactly N/n_C nodes to each community. Each node receives a random number of open connections drawn from a Poisson distribution with average $\bar{k}$. We then classify each of these connections as either a within-community or an inter-community stub with probabilities $1-\epsilon$ and ϵ, respectively. Links are finally created by matching stubs. Within-community (between-community) stubs are matched with each other according to a configuration model. We eventually discard self-links, multiple links between any pair of nodes and unmatched stubs. For large networks, the number of discarded stubs is usually negligible compared to the number of links. Notice that this algorithm enables us to independently set both the degree distribution and the strength of the community structure.

Simulating spread of concurrent diseases on networks

Simulations occur in discrete time. During each time step we check first for possible infection events caused by infected nodes and then for recovery events. Every infected node tries to transmit the disease(s) it is carrying to each of its neighbours. Each naive susceptible individual (compartment S) can get infected by pathogen A with probability $1-(1-\alpha)^{n_A}$, where n_A is the number of its susceptible neighbours carrying A. At the same time, a naive susceptible can also be infected by either B_1 or B_2. To avoid co-infections with B_1 and B_2, we loop over the neighbours of the naive node in a random order, checking for each infectious neighbour node if infection occurs or not (according to the corresponding infection probability, i.e. either β_1 or β_2) and stopping iteration at the first successful infection event. Transmission with either B_1 or B_2 can

occur independently from A, thus direct transitions from S to either D_1 or D_2 compartments are allowed. Secondary infections are implemented in a similar way.

For convenience, the simulation time step Δt was taken as the time unit. To avoid possible spurious effects due to time discretisation we set the infectious duration to be longer than the time step, i.e. $\mu^{-1} = 20\Delta t$. During each time step, infected individuals recover from each of the diseases they are carrying with probability μ. As a consequence a doubly infected individual can turn into a fully susceptible individual with probability μ^2. Individuals cannot recover during the same time step they got infected.

For each simulation, we initially choose 100 nodes for each pathogen and set them infected with that pathogen. In the Erdős-Rényi case initially infected nodes are chosen at random, whereas in the case of modular networks the infected seeds are chosen at random within the community where a particular pathogen is seeded. We stop simulations 400 time steps after either B_1 or B_2 becomes extinct, or, alternatively, after reaching the maximum simulation time T_{max}. The former stopping condition is dictated by the need to discern simulations where A is able to persist from those where it becomes extinct right after extinction of either one of the B strains. When any of the stopping conditions is met, we check which pathogens have survived and the corresponding prevalence. To reconstruct the phase diagram of figures 6 and 7 of the main paper we have 500 and 140 simulations for any given point in the parameter space for the Erdős-Rényi and modular networks respectively.

2 Results

2.1 Equilibria and stability analysis for the well-mixed system

Here we enumerate fixed points and study the stability of each equilibrium point by finding the eigenvalues of the corresponding Jacobian matrix $\mathcal{J}$. Because total density is conserved, the effective number of independent equations can be reduced from 6 to 5. Therefore $\mathcal{J}$ is a 5x5 matrix. In the following we eliminate A exploiting total density conservation and consider S, B_1, B_2, D_1, and D_2 as independent variables. A is kept as a placeholder for $1 - S - B_1 - B_2 - D_1 - D_2$. The general form of the Jacobian is given by:

$$\begin{pmatrix} \alpha(S^* - X_A^*) - 1 - \beta_1 X_1^* - \beta_2 X_2^* & S^*(\alpha - \beta_1) & S^*(\alpha - \beta_2) & -1 - \beta_1 S^* & -1 - \beta_2 S^* \\ c_1 \alpha B_1^* + \beta_1 X_1^* & c_1 \alpha (B_1^* - X_A^*) + \beta_1 S^* - 1 & c_1 \alpha B_1^* & 1 + \beta_1 S^* & 0 \\ c_2 \alpha B_2^* + \beta_2 X_2^* & c_2 \alpha B_2^* & c_2 \alpha (B_2^* - X_A^*) + \beta_2 S^* - 1 & 0 & 1 + \beta_2 S^* \\ -c_1(\alpha B_1^* + \beta_1 X_1^*) & c_1 \beta_1 (A^* - X_1^*) + c_1 \alpha (X_A^* - B_1^*) & -c_1(\alpha B_1^* + \beta_1 X_1^*) & c_1 \beta_1 (A^* - X_1^*) - 2 & -c_1 \beta_1 X_1^* \\ -c_2(\alpha B_2^* + \beta_2 X_2^*) & -c_2(\alpha B_2^* + \beta_2 X_2^*) & c_2 \beta_2 (A^* - X_2^*) + c_2 \alpha (X_A^* - B_2^*) & -c_2 \beta_2 X_2^* & c_2 \beta_2 (A^* - X_2^*) - 2 \end{pmatrix} \quad (1)$$

1. **Disease free state**: $S^* = 1$.

For this equilibrium $\mathcal{J}$ takes the form of an upper triangular matrix. Therefore the eigenvalues can be read off immediately since they coincide with the diagonal elements. In particular: $\lambda \in \{\alpha - 1, \beta_1 - 1, \beta_2 - 1, -2, -2\}$. Stability is therefore ensured if and only if all base transmission rates are smaller than 1.

2. **Pathogen A only** ($[A]$): $S^* = 1/\alpha, A^* = 1 - 1/\alpha$, which is feasible if and only if $\alpha > 1$.

   In this case one eigenvalue can be found immediately by inspection and it is equal to $1 - \alpha$, which is always negative when this equilibrium is feasible (i.e. $\alpha > 1$). The rest of the Jacobian matrix takes a 2x2 block diagonal form with diagonal blocks $J_i^{[A]}$ ($i = 1, 2$) given by:

$$J_i^{[A]} = \begin{pmatrix} -1 + c_i(1-\alpha)\beta_i/\alpha & 1 + \beta_i/\alpha \\ c_i(\beta_i + \alpha)(1 - \alpha^{-1}) & -2 + c_i\beta_i/\alpha \end{pmatrix}, \tag{2}$$

   whose eigenvalues can be determined by considering the matrix $\Gamma(\lambda) = J_i^{[A]} - \lambda I_2$, where I_n is the nxn identity matrix. We subtract the second column of Γ from the first column obtaining a new matrix Γ'. By construction $\det(\Gamma) = \det(\Gamma')$. Therefore, the characteristic polynomial is the same. Now, however, the latter polynomial already appears in a factorized form, yielding the eigenvalues $\lambda = -2 - c_i(\alpha - 1)$, which is always negative, and $\lambda = -1 + \beta_i c_i(1 - \alpha^{-1}) + \beta/\alpha$, which is negative if and only if $\beta_i < \dfrac{\alpha}{1 + c_i(\alpha - 1)}$. For $\alpha > 1$ (condition for the solution to be feasible, as written above), this is never true if either one of B_i is super-critical.

3. **Strain $\mathbf{B_i}$ only** ($[B_i]$): $S^* = 1/\beta_i, B_i^* = 1 - 1/\beta_i$, which is feasible if and only if $\beta_i > 1$.

   In the following we will use the index i to refer to strain B_i while the index j will indicate the competitor. Here $\mathcal{J}$ can be broken down into a 2x2 upper triangular matrix and a 3x3 matrix. The former has eigenvalues -2 and $-1 + \dfrac{\beta_j}{\beta_i}$. Therefore, stability requires $\beta_i > \beta_j$. The remaining 3x3 matrix $J_3^{[B_i]}$ takes the form:

$$J_3^{[B_i]} = \begin{pmatrix} \alpha S^* - 1 - \beta_i B_i^* & \alpha S^* - 1 & -2 \\ \alpha c_i B_i^* + \beta_i - 1 & \alpha c_i B_i^* & 2 \\ -c_i(\alpha + \beta_i)B_i^* & -c_i(\alpha + \beta_i)B_i^* & -2 - \beta_i c_i B_i^* \end{pmatrix}, \tag{3}$$

   which can be easily diagonalized by considering the matrix $\Gamma(\lambda) = J_3^{[B_i]} - \lambda I_3$ and performing the following row operations: first add its first row to its second row, then add the second row to its third row; the first row is left unchanged. This procedure enables writing down the characteristic polynomial in an easy-to-factorize form, yielding the eigenvalues $\lambda = 1 - \beta_i$, $\lambda = -2 - c_i(\beta_i - 1)$ and $\lambda = \alpha c_i B_i^* - 1 + \alpha\beta_i^{-1}$. The former two are always

negative when this equilibrium is feasible, while the latter is negative if and only if $\alpha < \dfrac{\beta_i}{1 + c_i(\beta_i - 1)}$, which is never true if the spreading of A is super-critical.

4. A **and** $\mathbf{B_i}$ **syndemic** ($[A\&B_i]$): By using the equilibrium conditions $\dot{X}_i = 0$ and $\dot{X}_A = 0$,
$$\beta_i(S^* + c_i A^*) - 1 = 0, \tag{4}$$
and
$$\alpha(S^* + c_i B_i^*) - 1 = 0, \tag{5}$$
we find that $B_i^* = \dfrac{1 - \alpha S^*}{c_i \alpha}$ and $A^* = \dfrac{1 - \beta_i S^*}{c_i \beta_i}$. By exploiting density conservation, i.e. $D_i^* = 1 - S^* - A^* - B_i^*$, we can express every variable in terms of S^*. The latter is determined by a quadratic polynomial whose roots are given by:
$$S^*_{\pm} = \frac{1 \pm \sqrt{1 - \dfrac{4}{c_i \beta_i \alpha}\left(1 - \dfrac{1}{c_i}\right)}}{2\left(1 - \dfrac{1}{c_i}\right)}\,, \tag{6}$$
which exists if $\alpha\beta_i > 4(c_i - 1)/c_i^2$.

Here $\mathcal{J}$ can be broken down into a 2x2 and a 3x3 matrices. The smaller matrix $J_2^{[A\&B_i]}$ is given by:
$$J_2^{[A\&B_i]} = \begin{pmatrix} \beta_j S^* - \alpha c_j X_A^* - 1 & 1 + \beta_j S^* \\ -c_j(\alpha X_A^* + \beta_j A^*) & -2 - \beta_j c_j A^* \end{pmatrix}, \tag{7}$$
which can be easily diagonalised by using row/column operations, yielding the eigenvalues $\lambda = -2 - c_j \alpha X_A^*$ and $\lambda = -1 + \beta_j(S^* + c_j A^*)$. The former is always negative while the latter corresponds to the asymptotic growth rate of X_j. This is the only eigenvalue in which parameters β_j, c_j, which pertain to the competitor strain, appear.

$J_3^{[A\&B_i]}$ instead takes the form:
$$\begin{pmatrix} \alpha(S^* - X_A^*) - \beta_i X_i^* - 1 & S^*(\alpha - \beta_i) & -1 - \beta_i S^* \\ c_i \alpha B_i^* + \beta_i X_i^* & c_i \alpha(B_i^* - X_A^*) - 1 + \beta_i S^* & 1 + \beta_i S^* \\ c_i(\alpha B_i^* + \beta_i X_i^*) & c_i \alpha(X_A^* - B_i^*) + c_i \beta_i(A^* - X_i^*) & -2 + c_i \beta_i(A^* - X_i^*) \end{pmatrix}. \tag{8}$$

Although the spectrum of $J_3^{[A\&B_i]}$ cannot be determined analytically, we can still gain some insight about stability conditions by studying its characteristic polynomial. We do so by first computing $\Gamma(\lambda) = J_3^{[A\&B_i]} - \lambda I_3$. We then consider the matrix Γ' obtained by adding the first row of Γ to the second row and adding the second row to the third row. The characteristic equation takes the form $P(\lambda) = \lambda^3 + a_2\lambda^2 + a_1\lambda + a_0 = 0$. Now, the

Routh-Hurwitz criterion states that in order for all $P(\lambda)$'s roots to have a negative real part, the following conditions must be satisfied: $a_2 > 0$, $a_0 > 0$ and $a_2 a_1 > a_0$. We find that:

$$a_2 = 2 + (c_i + 1)(\alpha X_A^* + \beta_i X_i^*), \tag{9}$$

$$a_1 = \alpha\beta_i c_i^2 X_i^* X_A^* + c_i(2+\alpha X_A^* + \beta_i X_i^*)(\alpha X_A^* + \beta_i X_i^*) + (1-c_i)(\alpha^2 X_A^* + \beta_i^2 X_i^*)S^* \tag{10}$$

$$a_0 = \alpha\beta_i c_i^2 X_i^* X_A^* (\alpha + \beta_i)(1 - 2(1 - 1/c_i)S^*), \tag{11}$$

so $a_2 > 0$ always. Substituting $S_\pm^*$ inside the definition of a_0 yields $a_0(S_+^*) < 0$ and $a_0(S_-^*) > 0$. Therefore according to the Routh-Hurwitz criterion the solution S_+^* is never found to be stable. S_-^* is, instead, stable when the condition $a_2 a_1 > a_0$ is satisfied. The feasibility of $[A\&B_i]$ state can be checked numerically as well by requiring that $S^*, A, B_i^*, D_i^* > 0$. In particular, the condition $\alpha\beta_i > 4(c_i - 1)/c_i^2$ ensures that S_-^* is real and positive and explains the vertical boundary delimiting the $[A\&B_2]$ stable region in figure 2b in the main manuscript.

5. **All strains coexist** We first consider the equilibrium conditions $\dot{X}_i = 0$, $i = 1, 2$, given by Eq. (4), which allow us to obtain S^* and A^*:

$$S^* = \frac{c_2\beta_2 - c_1\beta_1}{\beta_1\beta_2(c_2 - c_1)},$$

$$A^* = \frac{\beta_2 - \beta_1}{\beta_1\beta_2(c_1 - c_2)}.$$

Notice that if $\beta_2 > \beta_1$, then we need $c_1 > c_2$ and $c_1\beta_1 > c_2\beta_2$ for this fixed point to be feasible. After some algebra one can obtain a quadratic equation for B_1^*:

$$\begin{aligned}
&\Big(1 + \beta_2 S^*\Big)\Big(1 - S^* - A^*\Big) + c_2^{-1}\Big(S^* - \alpha^{-1}\Big)\Big(1 + \alpha S^* + \alpha c_2(1 - S^*)\Big) + \\
&B_1^*\Bigg\{ -1 - \beta_2 S^* - \frac{1 + \beta_2 S^*}{1 + \beta_1 S^*}\Big(1 - c_1/c_2 + \alpha c_1 - (\beta_1 + \alpha c_1(1 - c_2^{-1}))S^*\Big) + \\
&\Big(1 - \alpha S^*\Big)\Big(1 - c_1/c_2\Big) + c_1/c_2\Big(1 + \alpha S^* + \alpha c_2(1 - S^*)\Big)\Bigg\} + \\
&B_1^{*2}\alpha c_1\Big(c_1/c_2 - 1\Big)\left(1 - \frac{1 + \beta_2 S^*}{1 + \beta_1 S^*}\right) = 0.
\end{aligned} \tag{12}$$

Once the roots of the latter equations have been found, the remaining fractions can be computed using:

$$B_2^* = c_2^{-1}\Big(\alpha^{-1} - S^* - c_1 B_1^*\Big),$$

$$D_1^* = \frac{1 - \beta_1 S^* + \alpha c_1 \Big(1 - S^* - B_1^* - B_2^*\Big)}{1 + \beta_1 S^*} B_1^*,$$

$$D_2^* = 1 - S^* - A^* - B_1^* - B_2^* - D_1^*.$$

By numerically computing these equilibria, we found that they can be feasible under certain conditions on the parameters. The condition for their stability cannot be computed analytically. However, we can still evaluate the Jacobian at the fixed points and then compute its eigenvalues numerically and find that the equilibria are always unstable.

Additional results

References

[1] Vladimir Batagelj and Ulrik Brandes. Efficient generation of large random networks. *Phys. Rev. E*, 71(3):036113, March 2005.

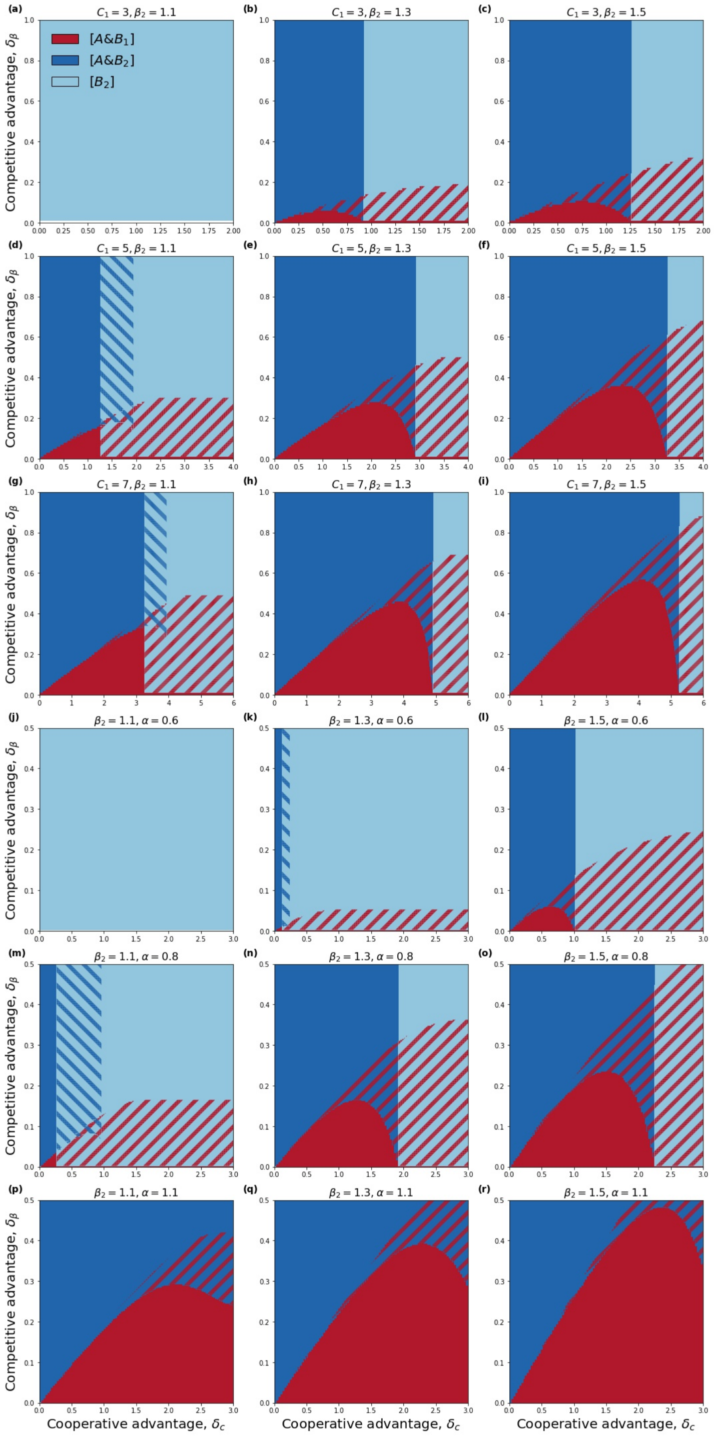

Figure S1: Phase diagram for different values of β_2, α and c_1. (a)-(i) $\alpha = 0.8$. (j)-(r) $c_1 = 4$. The range of β_1 includes values below 1.

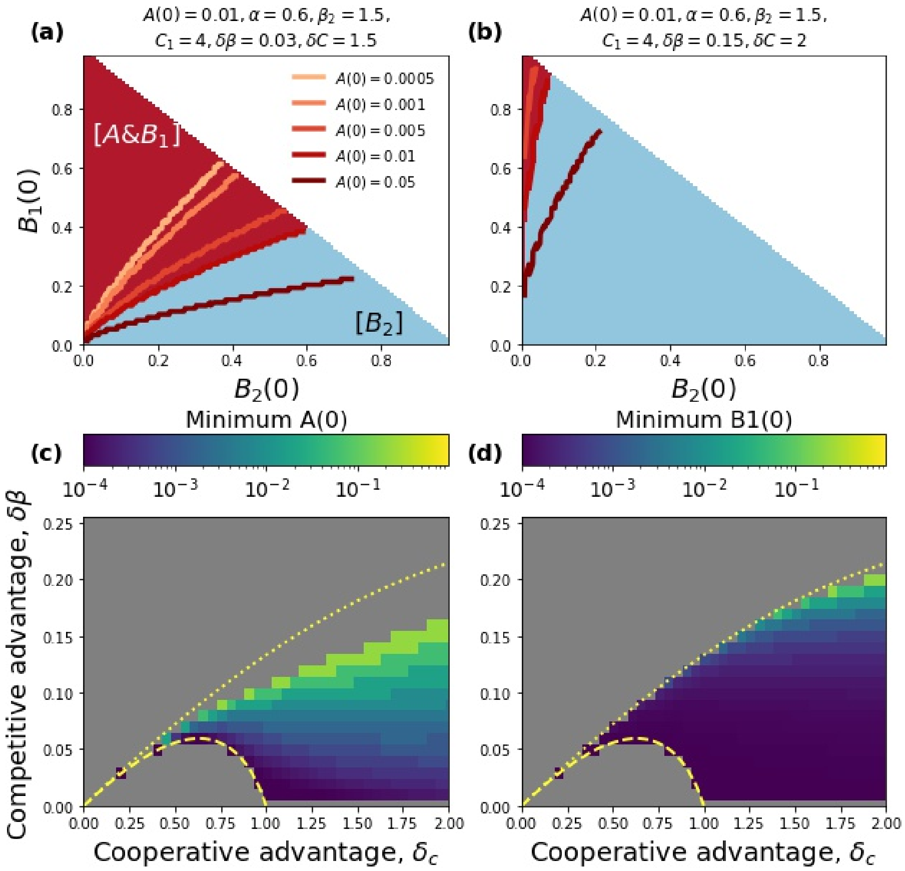

Figure S2: Initial conditions and mean-field dynamics. (a),(b) Final outcome as a function of $B_1(0)$ and $B_2(0)$ with $A(0) = 0.01$. Additional boundaries separating the two dominance regions and corresponding to different values of $A(0)$ are also showed. For each value of $A(0)$ we explore values of $B_1(0)$ and $B_2(0)$ in the simplex $0 \leq B_1(0) + B_2(0) \leq 1 - A(0)$. Parameters were: (a) $\delta_\beta = 0.03$, $\delta_c = 1.5$, (b) $\delta_\beta = 0.15$, $\delta_c = 2$. (c) Minimum amount of $A(0)$ required in order for B_1 to win as a function of δ_β and δ_c, given that $B_1(0) = B_2(0) = 0.001$. (d) Minimum amount of $B_1(0)$ required in order for B_1 to win as a function of δ_β and δ_c, given that $A(0) = 0.01$ and $B_2(0) = 0.001$. Grey regions in (c,d) correspond to either absence of bistability or to parameter choices for which B_1 never wins. This figure shows that a sufficiently large advantage in terms of initial conditions can lead to B_1 winning the competition in the bistable region. Other parameters are the same as in figure 2a of the main manuscript.

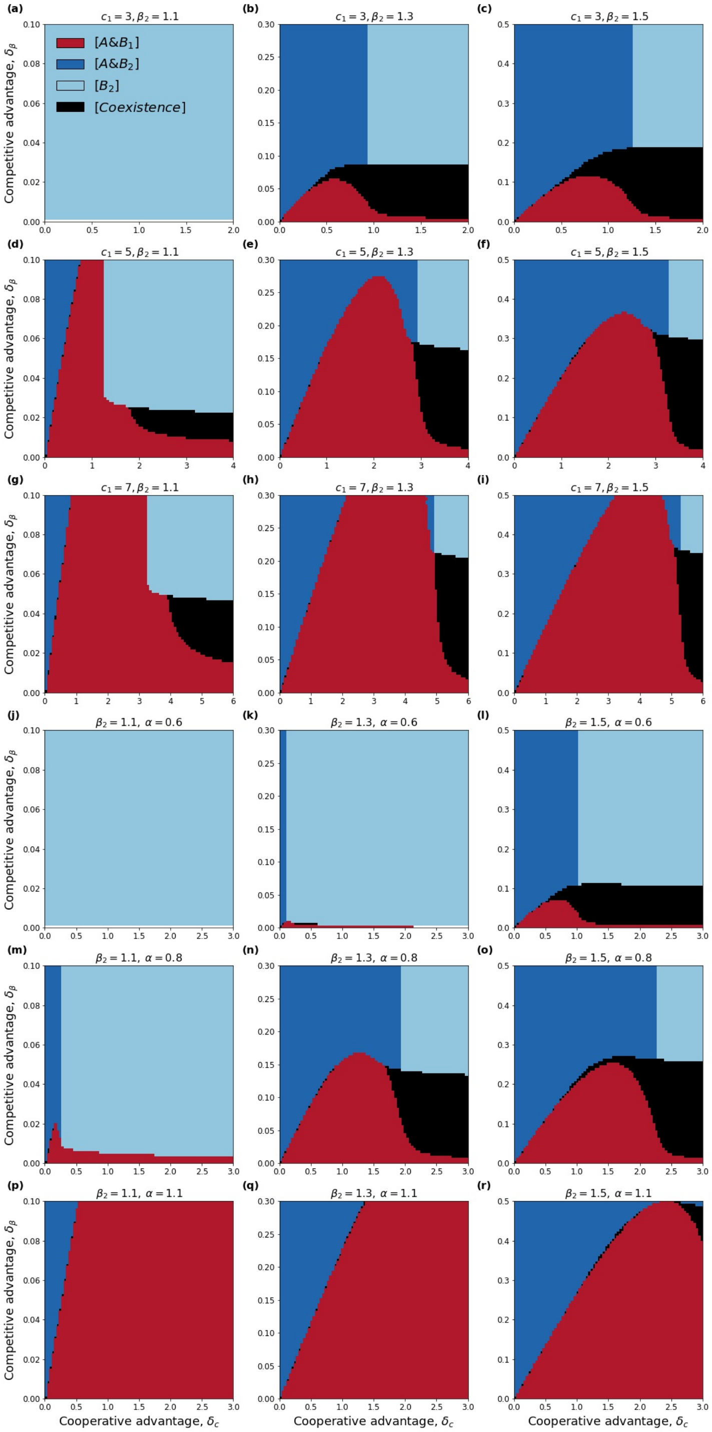

Figure S3: Equilibrium configuration for two interacting communities for different values of $\boldsymbol{\beta_2}$, $\boldsymbol{\alpha}$ and c_1. (a)-(i) $\alpha = 0.8$. (j)-(r) $c_1 = 4$. Initial conditions are as in figure 4c of the main paper. Here we have considered values of δ_β and δ_c such that β_1, β_2, c_1 and c_2 are all larger than unity. Increasing values of α have a positive effect on the persistence of B_1.

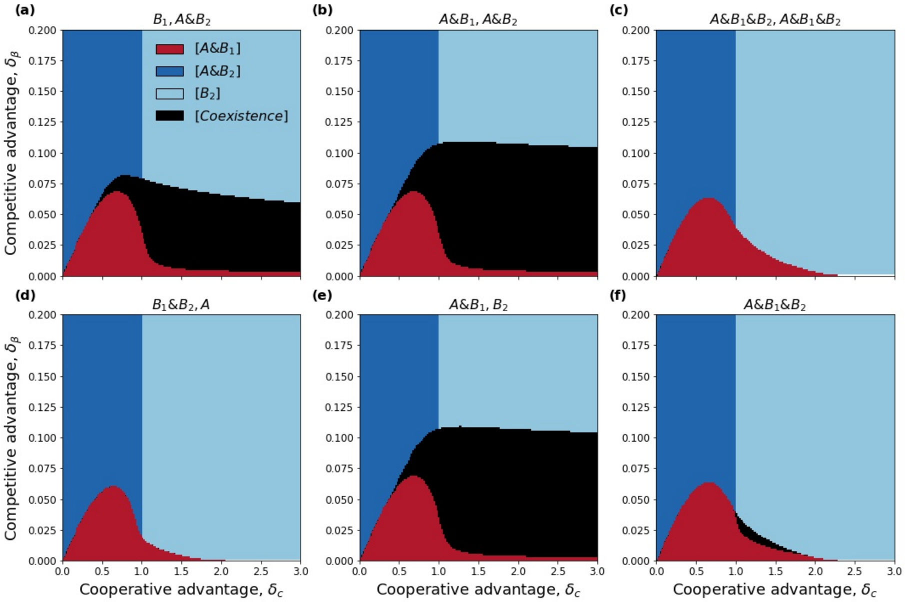

Figure S4: Role of initial conditions in mean-field dynamics with communities. Final outcome as a function of δ_c and δ_β for different initial conditions. The title of each panel indicates in which communities each pathogen is seeded into; the initial density of a pathogen in the community it is seeded into is set to 0.01. Other parameters are as in figure 4 of the main manuscript.

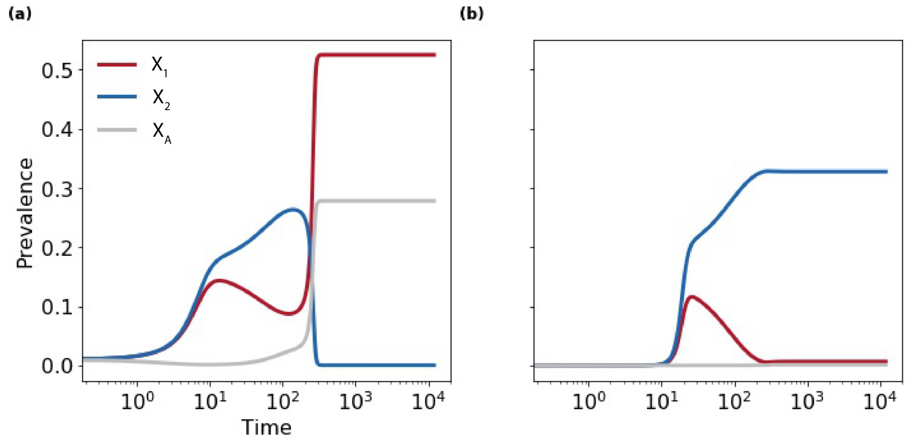

Figure S5: Dynamics with two strains seeded in the same community. Dynamical trajectories of B_1's, B_2's and A's total prevalence (in red, blue and gray respectively) in community 1 (a) and 2 (b). Trajectories were obtained numerically by starting from $B_1^{(1)}(t=0) = B_2^{(1)}(t=0) = A^{(1)}(t=0) = 0.01$. Around $t = 10$, B_2 takes over community 2 as a results of its advantage in transmissibility. At this point B_1's prevalence declines until A gives rise to a new outbreak; the latter ultimately leads to a B_1 dominance in the first community. Here we set $\delta_\beta = 0.025$, $\delta_c = 1.2$ and $\epsilon = 0.0002$. Other parameters are as in figure 2a.

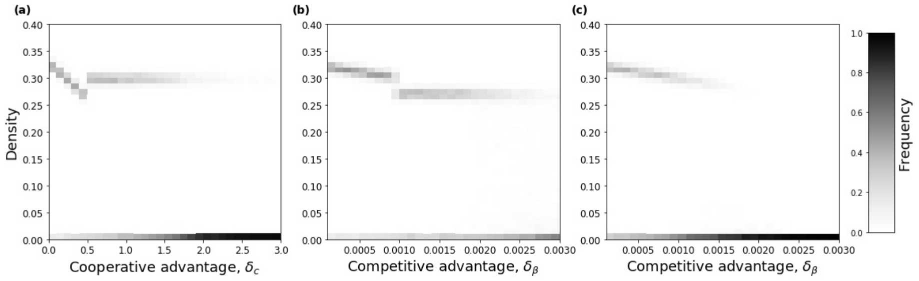

Figure S6: Final-state density of X_A for stochastic simulations on ER networks. (a-c) show the probability of the final state for a given value of X_A. Here we do not differentiate between which final state is attained, i.e. which pathogen persists. (a) has been obtained for $\delta_\beta = 0.001$, while (b) and (c) have been obtained for $\delta_c = 0.4$ and $\delta_\beta = 1.5$, respectively. In (a) we can observe two types of transitions in the behaviour of X_A: the sharp transition at $\delta_c = 0.5$ corresponds to a leap from state $[A\&B_2]$ to state $[A\&B_1]$. As δ_c is further increased, extinction of A becomes increasingly probable as the state $[B_2]$ is reached with a higher frequency. This transition is, however, gradual and around $\delta_c = 1.3$ a crossover is reached where states $[A\&B_1]$ and $[B_2]$ are reached with equal probability. Other parameters are as in figure 6.
